# Structure based approach for understanding organism specific recognition of protein-RNA complexes

**DOI:** 10.1186/s13062-015-0039-8

**Published:** 2015-03-07

**Authors:** Raju Nagarajan, Sonia Pankaj Chothani, Chandrasekaran Ramakrishnan, Masakazu Sekijima, M Michael Gromiha

**Affiliations:** Department of Biotechnology, Bhupat Jyoti Metha School of Biosciences, Indian Institute of Technology Madras, Chennai, 600036 Tamilnadu India; Philips Research North America, 345 Scarborough Road, Briarcliff Manor, NY 10510 USA; Global Scientific Information and Computing Center (GSIC), Tokyo Institute of Technology, 2-12-1 Ookayama, Meguro-ku, Tokyo 152-8550 Japan

**Keywords:** Protein-RNA complex, Organism specificity, Binding specificity, Binding motifs, Propensity, Recognition mechanism

## Abstract

**Background:**

Protein-RNA interactions perform diverse functions within the cell. Understanding the recognition mechanism of protein-RNA complexes has been a challenging task in molecular and computational biology. In earlier works, the recognition mechanisms have been studied for a specific complex or using a set of non–redundant complexes. In this work, we have constructed 18 sets of same protein-RNA complexes belonging to different organisms from Protein Data Bank (PDB). The similarities and differences in each set of complexes have been revealed in terms of various sequence and structure based features such as root mean square deviation, sequence homology, propensity of binding site residues, variance, conservation at binding sites, binding segments, binding motifs of amino acid residues and nucleotides, preferred amino acid-nucleotide pairs and influence of neighboring residues for binding.

**Results:**

We found that the proteins of mesophilic organisms have more number of binding sites than thermophiles and the binding propensities of amino acid residues are distinct in *E. coli*, *H. sapiens*, *S. cerevisiae*, thermophiles and archaea. Proteins prefer to bind with RNA using a single residue segment in all the organisms while RNA prefers to use a stretch of up to six nucleotides for binding with proteins. We have developed amino acid residue-nucleotide pair potentials for different organisms, which could be used for predicting the binding specificity. Further, molecular dynamics simulation studies on aspartyl tRNA synthetase complexed with aspartyl tRNA showed specific modes of recognition in *E. coli, T. thermophilus and S. cerevisiae*.

**Conclusion:**

Based on structural analysis and molecular dynamics simulations we suggest that the mode of recognition depends on the type of the organism in a protein-RNA complex.

**Reviewers:**

This article was reviewed by Sandor Pongor, Gajendra Raghava and Narayanaswamy Srinivasan.

**Electronic supplementary material:**

The online version of this article (doi:10.1186/s13062-015-0039-8) contains supplementary material, which is available to authorized users.

## Background

Protein-RNA interactions play critical roles in determining the structure of the ribosome and spliceosome, and gene expression. The interaction of proteins with RNA has been generally explained using different types of motifs such as Arginine rich motif, RNA recognition motif, GXXG motif, double stranded RNA binding motif, tetra loops (GX[GA]A) in RNA and so on [1]. The recognition mechanisms of protein-RNA complexes and their functional importance have been mainly elucidated by three-dimensional structure determination of protein-RNA complexes [2] along with other molecular biology experiments such as site directed mutagenesis, fluorescence resonance energy transfer (FRET) imaging, etc. The structures of protein-RNA complexes have been effectively used for identifying the binding sites using distance based criteria, solvent accessibility based method and energy based approach [3-5].

The availability of protein-RNA complex structures in PDB [6] has enabled researchers to develop secondary databases [7,8] and to analyze the binding sites in terms of atomic contacts, amino acid composition, preference of residues, secondary structures, solvent accessibility, electrostatic interactions, hydrophobic contacts, hydrogen bonding, cation-π, stacking and van der Waals interactions [3,9,10]. The results obtained from the structural analysis of protein-RNA complexes have been successfully utilized for understanding their recognition mechanism and predicting the binding sites. Further, Pietal et al. developed a method for visualizing and analyzing contact and distance maps for protein-RNA complex structures [11]. Recently, Fornes et al. reviewed the applications of knowledge-based potentials for evaluating the models of protein-RNA interactions along with other complexes [12].

On the other hand, several methods based on machine learning techniques have been proposed for identifying the binding sites in protein-RNA complexes. These methods utilize different features such as side chain pKa, hydrophobicity index, molecular mass, evolutionary conservation, predicted secondary structure, solvent accessibility and PSSM profiles [13-17]. Recently, Nagarajan and Gromiha (2014) analyzed the performance of various methods for identifying the binding sites in protein-RNA complexes based on protein structural class, fold, family, superfamily, function, RNA structure, and conformation.

The structural analysis of protein-RNA complexes and prediction methods mainly utilize non-redundant set of complexes for avoiding bias in the analysis. This assumption is based on the fact that the structure and function of protein-RNA complexes are similar if the protein sequences are homologous to each other. We have addressed this issue by analyzing the binding sites of same protein-RNA complexes belonging to different organisms in which the protein sequences are redundant among themselves. We have developed a dataset of protein-RNA complexes from different organisms with high sequence identity and identified the binding sites. The binding sites have been analyzed in terms of binding propensity, amino acid-nucleotide pair preference, binding motif etc. We have found that the proteins of mesophiles contain more binding sites than thermophiles and the binding propensities of amino acid residues are distinct in each organism. Positively charged residues have high preference in *E. coli*, aromatic residues are preferred in *S. cerevisiae*, polar residues in thermophiles, Gly and Trp in *H. sapiens* and a mixed combination of residues in archaea. The binding propensities of polar residues showed high variability among different organisms at conserved positions. The analysis on the preference of amino acid-nucleotide residue pairs revealed that the amino acid residues prefer to pair with cytosine in *E. coli* though the preference is mainly with adenosine in *H. sapiens* and *S. cerevisiae*. Thermophiles and archaea showed high preference to interact with cytosine and uracil, respectively. Further, molecular dynamics simulations studies on aspartyl tRNA synthetase complexed with aspartyl tRNA (AspRS-tRNA^Asp^) indicated distinct modes of recognition in different organisms.

## Methods

### Dataset

We have constructed 18 sets of protein-RNA complexes belonging to different organisms. The datasets have been obtained by carefully searching such complexes in PDB [6] with the following criteria: (i) structures of protein-RNA complexes are known for at least two organisms, (ii) protein should have a minimum of 30 residues, (iii) RNA should have at least 5 nucleotides and (iv) the sequence identity of proteins among these complexes is more than 25%. The list of 18 sets of complexes along with their structural similarity (RMSD score) and sequence identity have been summarized in Table 1. The crystallization temperature is 100 K for most of the complexes (>90%) and all of them are expressed in E. coli [6].

**Table 1:** **List of protein-RNA complexes used in the present study**

**Complex**	**Organism**	**PDB code**	**RMSD (Å)**	**Sequence identity (%)**	**Binding site residues**	
					**Protein (%)**	**RNA (%)**
Elongation Factor TU	*E. coli*	1OB2:A	1.4	71	7.63	22.37
	*T. aquaticus*	1OB5:A			9.14	20.78
Leucyl-tRNA synthetase	*E. coli*	4ARC:A	1-2: 1.9	1-2: 45	4.09	25.29
	*T. thermophilus*	2BTE:A	1–3: 2.5	1–3: 27	3.08	22.89
	*P. horikoshii*	1WZ2:A	2–3: 1.6	2–3: 29	3.21	18.18
Retinoic acid inducible protein I	*A. platyrhynchos*	4A36:A	2.6	59	4.14	28.95
	*H. sapiens*	3TMI:A			3.17	42.86
Glutamyl-tRNA synthetase	*T. maritima*	3AKZ:A	2.3	41	9.65	33.78
	*T. thermophilus*	1N78:A			9.19	30.67
Aspartyl-tRNA synthetase	*E. coli*	1IL2:A	1-2: 2.1	1-2: 49	7.12	32.00
	*T. thermophilus*	1EFW:A	1–3: 2.2	1–3: 28	3.97	20.55
	*S. cerevisiae*	1ASY:A	2–3: 2.3	2–3: 30	8.57	26.67
Signal recognition particle	*H. sapiens*	1MFQ:C	1-2: 2.7	1-2: 36	9.30	6.25
	*M. jannaschii*	2V3C:C	1–3: 5.4	1–3: 32	5.79	18.48
	*S. solfataricus*	1QZW:A	2–3: 1.29	2–3: 48	2.50	11.70
ATP dependent RNA helicase	*H. sapiens*	3G0H:A	1.1	51	5.19	100.00
	*S. cerevisiae*	3PEY:A			5.57	83.33
Tyrosyl-tRNA synthetase	*S. cerevisiae*	2DLC:X	1-2: 1.7	1-2: 35	2.79	5.26
	*M. jannaschii*	1J1U:A	1–3: 2.7	1–3: 26	4.58	6.49
	*T. thermophilus*	1H3E:A	2–3: 2.2	2–3: 27	4.63	16.28
Probable exosome complex exonuclease 1	*A. fulgidus*	3M7N:D	0.9	59	2.33	33.33
	*P. abyssi*	2PO1:A			3.21	40.00
50S ribosomal protein L7Ae	*A. fulgidus*	1RLG:A	0.8	60	13.45	36.00
	*M. jannaschii*	1SDS:A			17.09	23.33
60S ribosomal protein L7	*S. cerevisiae*	3O5H:G	1.9	47	1.23	1.65
	*T. thermophila*	4A1C:V			2.51	5.00
STAR family quaking protein	*C. elegans*	4JVY:A	2.8	58	10.20	71.43
	*H. sapiens*	4JVH:A			8.61	63.64
Retinoic acid inducible protein I	*A. platyrhynchos*	4A2X:A	1.7	55	3.05	14.29
	*H. sapiens*	3NCU:A			7.46	25.00
Arginyl-tRNA synthetase	*S. cerevisiae*	1F7U:A	2.6	29	7.25	32.89
	*P. horikoshii*	2ZUF:A			6.20	32.05
Pumilio mRNA binding factor	*S. cerevisiae*	3 K49:A	1.1	45	9.49	90.00
	*H. sapiens*	2YJY:A			7.43	90.00
tRNA pseudouridine synthase B	*E. coli*	1K8W:A	1.8	34	11.31	54.55
	*T. maritime*	1R3E:A			10.36	70.59
Signal recognition particle 19 kDa protein	*M. jannaschii*	1LNG:A	2.1	34	35.82	16.49
	*S. solfataricus*	3KTW:A			22.94	19.79
Phenylalanyl-tRNA synthetase	*T. thermophiles*	2IY5:A	2.1	31	3.14	7.89
	*H. sapiens*	3TUP:A			4.58	25.00

### Identification of binding site residues

Generally, binding site residues in protein-RNA complex structures have been identified with three different criteria: (i) distance between contacting atoms in protein and RNA using a specific cut-off value [18,19], (ii) reduction of solvent accessibility upon binding [20] and (iii) inter-residue interaction energy [21]. We have used the distance based approach to identify the binding site residues/nucleotides for the considered protein-RNA complexes. In this method, we have calculated the distance between the heavy atoms in protein and RNA. Two atoms (one in protein and another in RNA) are considered to be interacting with each other if the distance between them is less than 3.5 Å [5]. The respective residues and nucleotides are treated as binding site residues and nucleotides.

### Binding propensity

The binding propensity for the 20 amino acid residues and 4 nucleotides present in protein-RNA complexes has been calculated using following procedure [21-23]:

(i) We computed the frequency of occurrence of amino acid residues (nucleotides) in binding sites (f_b_) and in the protein (RNA) as a whole (f_t_). The binding propensity (P_bind_) is calculated using the equation:

1 $$ {\mathrm{P}}_{\mathrm{b}\mathrm{ind}}\left(\mathrm{i}\right)={\mathrm{f}}_{\mathrm{b}}\left(\mathrm{i}\right)*100/{\mathrm{f}}_{\mathrm{t}}\left(\mathrm{i}\right) $$

where, i represents each of the 20 amino acids and 4 nucleotides.

(ii) The binding propensity was normalized with the percentage of binding site residues in the considered protein-RNA complexes. The normalization factor (Norm) was calculated as follows:

2 $$ \mathrm{Norm}={\mathrm{f}}_{\mathrm{b}}/{\mathrm{f}}_{\mathrm{t}} $$

where, f_b_ is the total binding residues (nucleotides) and f_t_ is the total number of residues (nucleotides) in the considered protein-RNA complexes.

(iii)The normalized binding propensity (Pnorm_bind_) for the 20 amino acid residues and 4 nucleotides of RNA present in protein-RNA complexes was developed as follows:

3 $$ {\mathrm{P}}_{\mathrm{normbind}}\left(\mathrm{i}\right)={\mathrm{P}}_{\mathrm{bind}}\left(\mathrm{i}\right)/\mathrm{Norm} $$

The comparison among specific pairs of protein-RNA complexes from different organisms have been carried out using the normalized propensity of all and conserved residues along with the propensity of residues in five typical groups such as *E. coli*, *H. sapiens*, *S. cerevisiae*, thermophiles and archaea.

### Conservation of amino acid residues

We have evaluated the conservation of residues in each RNA binding protein using the server, Consurf [24] available at http://consurf.tau.ac.il/. We have selected JTT evolutionary substitution model for amino acid replacements and Bayesian method for computing the score. Consurf compares the sequence of a protein chain with the proteins deposited in Uniprot and displays the sequences that are homologous to the given protein sequence. All the sequences that were found to be evolutionarily related with a RNA binding protein chain within the dataset were subsequently analysed using multiple sequence alignment. These protein sequence alignments were used to classify all the residues in each RNA binding protein into 9 categories: highly variable (score: 1) to highly conserved (score: 9).

### Binding segments

The residues identified as binding sites have been studied in terms of binding segments. It is based on the number of consecutive binding residues in the amino acid sequences. For example, a 4-residue binding segment has a stretch of four consecutive binding residues. We have analyzed the binding segments with one, two, three, four, five, six and more than six residues. Similar analysis has also been carried out for nucleotides in RNA.

### Preference of amino acid-nucleotide pairs

The preference of amino acid-nucleotide pairs at the interface of protein-RNA complex in specific organism has been computed using the following equation [4]:

4 $$ {\mathrm{Pair}}_{\mathrm{org}}\left(\mathrm{i},\mathrm{j}\right)=\varSigma {\mathrm{N}}_{\mathrm{i}\mathrm{j}}/\left(\varSigma {\mathrm{N}}_{\mathrm{i}}+\varSigma {\mathrm{N}}_{\mathrm{j}}\right) $$

where i and j stands for the interacting residues and nucleotides in proteins and RNA, respectively. N_i,j_ is the number of interacting residues of type i in protein and j in RNA. *Σ*N_i_ and *Σ*N_j_ are the total number of residues and nucleotides i and j in protein and RNA, respectively.

The amino acid-nucleotide pair preference for each organism has been normalized with the preference of all protein-RNA complexes [Pair(i,j)] to obtain the propensity of amino acid-nucleotide pairs at the interface. It is given by

5 $$ \mathrm{Propen}\left(\mathrm{i},\mathrm{j}\right)={\mathrm{Pair}}_{\mathrm{org}}\left(\mathrm{i},\mathrm{j}\right)/\mathrm{Pair}\left(\mathrm{i},\mathrm{j}\right) $$

The propensity has been converted into potentials for the amino acid-nucleotide pairs using standard procedures [25].

6 $$ \mathrm{Potential}\left(\mathrm{i},\mathrm{j}\right)=-\mathrm{R}\mathrm{T}\  \ln\ \mathrm{Propen}\left(\mathrm{i},\mathrm{j}\right) $$

where R is the gas constant and T is the temperature.

### Influence of neighboring residues and motifs for binding with RNA

We have analyzed the influence of neighboring residues of binding sites using various aspects: (i) *B and B*, where * is any residue and B is a binding site residue. Further, the preferred tripeptide and trinucleotide motifs have been identified with a pattern, *B* [4,26]. As the number of combinations is high for tetrapeptides there will be no significant hits and hence we did not consider tetrapeptides in this work.

### Molecular dynamics simulations

We have analyzed the mode of recognition of tRNA^Asp^ by aspartyl tRNA synthetase (AspRS) in different organisms [27-29] using molecular dynamics simulations. The simulations were performed for 20 ns in an explicit water environment using ff99SB force field in AMBER suite [30-32]. The force field parameters of the modified tRNA bases were obtained from the Modifieds database [33]. Energy minimization and equilibrations were performed to remove the steric clashes and to set the temperature at 300 K and pressure at 1 atm using Berendsen thermostat coupling [34]. SHAKE algorithm [35] and Particle Mesh Ewald (PME) method [36] were employed to treat the hydrogen bonds and long range electrostatic interactions, respectively. Production runs (unrestrained) were carried out for 20 ns with 2 fs time step for each AspRs-tRNA^Asp^ complex. The binding free energy (ΔG°) calculations have been performed with MM-GB/SA method [37-39] for identifying the active site amino acids, which are strongly interacting with the tRNA^Asp^. The calculation of ΔG° for each residue has been carried out using pairwise decomposition with mmpbsa.py module [40].

## Results and discussion

### Percentage of binding site residues in protein-RNA complexes from different organisms

We have computed the percentage of binding site residues in all the considered protein-RNA complexes and the results obtained for different organisms are presented in Table 1. Our analysis showed that the percentage of binding site residues varies with organisms for the same protein-RNA complex. For example, the binding site residues in AspRS are 7.12%, 3.97% and 8.57% of total residues for *E. coli*, *T. thermophilus* and *S. cerevisiae,* respectively. On the other hand, the binding site nucleotides are 32.00%, 20.55% and 26.67%, respectively. These data reveal that the binding sites of thermophilic proteins are less than mesophiles both in protein and RNA; specifically, the differences in aspartyl tRNA synthetase are 3% and 11%, respectively. Similar trend is also observed in leucyl tRNA synthetase. This may be due to the fact that the residues in thermophiles are contributing towards the stability of proteins, whereas mesophiles show higher tendency to interact with RNA than thermophiles. In EF-Tu elongation factor, mesophilic *E. coli* has less number of binding residues though it has more number of binding nucleotides. Overall analysis reveals that the recognition depends on the organism for a protein-RNA complex.

### Binding propensity of residues in protein-RNA complexes from different organisms

**Figure 1:**
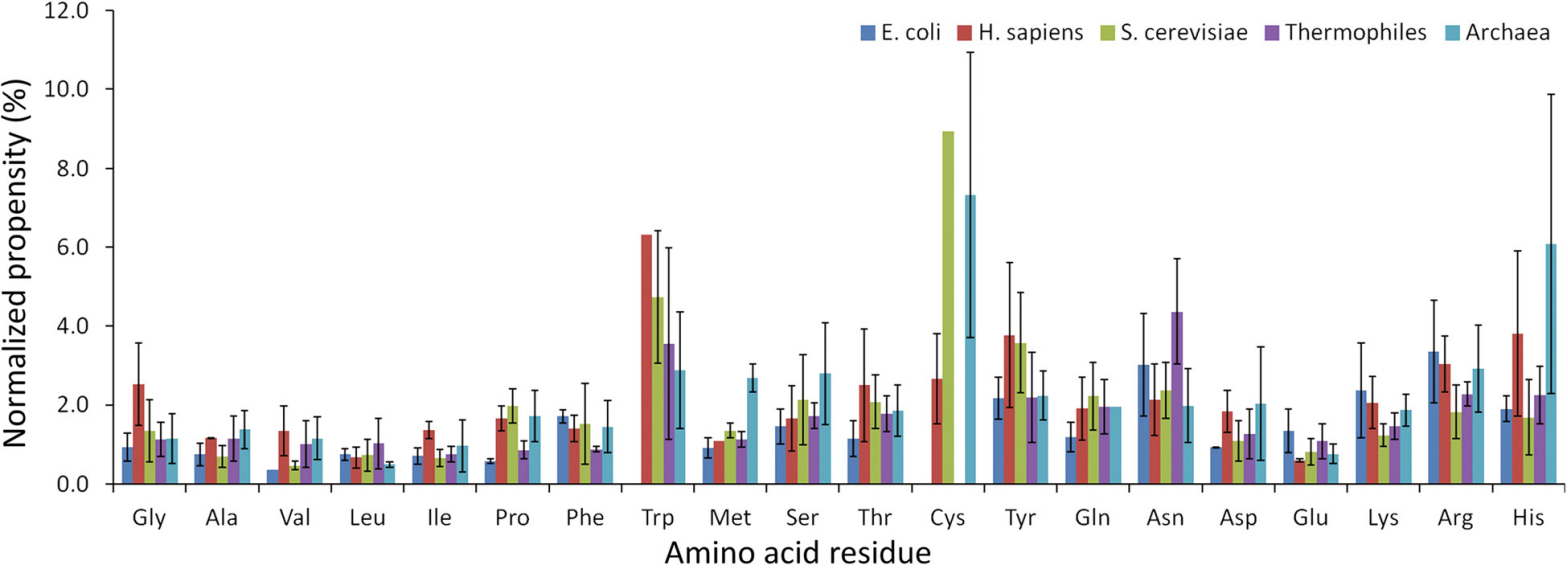
**Normalized binding propensity of amino acid residues in different organisms.**

We have computed the normalized binding propensity of all the 20 amino acid residues in different organisms (*E. coli*, *H. sapiens*, *S. cerevisiae*, thermophiles and archaea) and the results are shown in Figure 1. The analysis has been carried out on two aspects: in the first case, we have considered all the protein-RNA complexes in a single organism together and computed the average propensity and secondly, we have computed the propensity for each complex in an organism individually and computed the average and deviation. In this computation, residues with no binding sites were not taken into consideration. Noticeably, the trend is qualitatively similar in both results. We observed that the residues Ala, Val, Leu, Ile, Asp and Glu with the majority of hydrophobic residues have the normalized binding propensity of less than 2 and hence are not preferred at the binding sites. On the other hand, Ser, Tyr, Gln, Asn, Lys, Arg and His have the binding propensity of more than 2 in all the organisms showing their preferences at the interface. These results are similar to the binding propensity of residues obtained with energy based approach in a set of 81 protein-RNA complexes [4]. Interestingly, we noticed few differences in the binding propensity of residues among different organisms. Pro, Cys and Gln show higher preference in *S. cerevisiae* than other organisms. Lys, Arg and Phe are highly favored in E coli whereas Gly and Trp are preferred in *H. sapiens*. Asn shows high preference in thermophilic proteins although their overall composition is less than mesophilic ones [41]. Protein-RNA complexes from archaea are preferred with Ala, Pro, Met, Ser, Asp and His (Figure 1). In essence, the preference of amino residues at the interface of protein-RNA complexes is distinct in different organisms: positively charged residues in *E. coli*, aromatic residues in *S. cerevisiae*, polar residues in thermophiles, Gly and Trp in *H. sapiens* and a mixed combination of residues in archaea. These differences in binding sites residues among different organisms reflect their specific mode of recognition with RNA. Further, we have examined the statistical significance of the results and found that the p-value is less than 0.05.

### Binding propensity of nucleotides in different organisms of protein-RNA complexes

**Figure 2:**
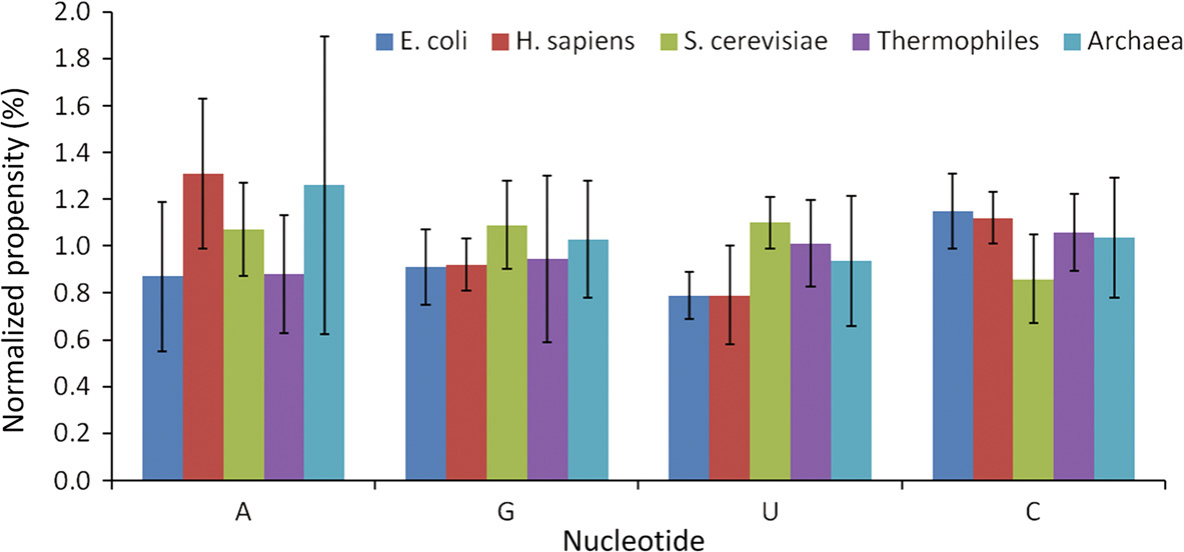
**Normalized binding propensity of nucleotides in different organisms.**

We have computed the normalized binding propensity of nucleotides in *E. coli*, *H. sapiens*, *S. cerevisiae*, thermophiles and archaea, and the results are presented in Figure 2. We observed that the propensity is high for adenine in *H. sapiens* and archaea, uracil in *S. cerevisiae* and cytosine in *E. coli* and thermophilies. Cytosine has the propensity of more than one in 4 of the 5 considered groups. The propensity of guanine lies between the propensities of other nucleotides in all organisms. This analysis also emphasizes different modes of recognition by different organisms. However, it is noteworthy that the difference in propensity among the four nucleotides in different organisms is less than that of 20 amino acid residues.

### Variations of binding propensities in conserved residues of protein-RNA complexes from different organisms

We have further analyzed the normalized binding propensities of amino acid residues at conserved positions of *E. coli*, *H. sapiens*, *S. cerevisiae*, thermophiles and archaea in protein-RNA complexes. We observed that the overall tendency of amino acid residues is similar for both conserved and other positions, and few residues showed remarkable differences in their propensities at the conserved binding sites. In *E. coli*, Glu has more preference for the binding sites of conserved positions compared to its propensity at all binding sites. Similar results were observed for Asn in *H. sapiens*, Glu and Lys in thermophiles and Lys in archaea. On the other hand, an opposite trend was observed for few other residues: Cys in *H. sapiens*, Trp in *S. cerevisiae*, Tyr in thermophiles, and Gln and His in archaea. These results indicate the role of residue conservation for the interactions between protein and RNA and specifically the influence of polar residues at conserved positions in different organisms of protein-RNA complexes.

### Influence of RNA base sequence on binding propensity

We have evaluated the influence of RNA base sequence on the binding propensity of amino acid residues in nucleotides. The lengths of RNA sequences are almost similar in all the complexes and the sequence identity varies in the range of 40-100% in most of the considered complexes. We have analyzed the nucleotide sequences at the binding sites in different pairs of protein-RNA complexes and observed that the binding preference is similar for all the nucleotides. Further, the change in propensities of amino acid residues is not uniform with the corresponding change in nucleotides. These analyses reveal that the influence of base sequence is not appreciable compared with amino acid sequences of protein-RNA complexes from different organisms. However, this effect can be extensively studied using systematic analysis on mutations and molecular dynamics simulations for deriving a conclusion.

### Binding segments in protein-RNA complexes belonging to different organisms

**Figure 3:**
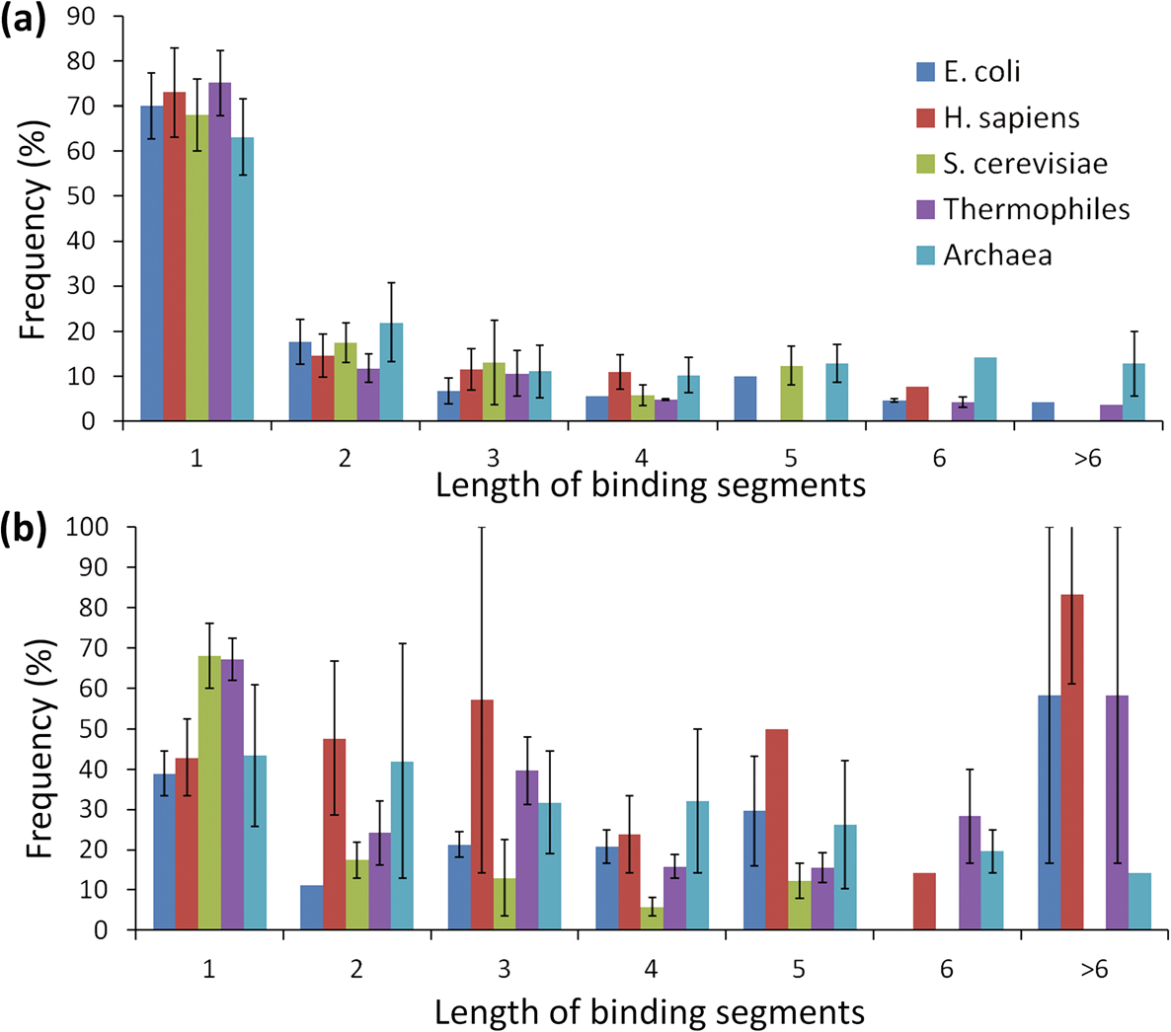
**Variation of binding segments in (a) proteins and (b) RNA.**

We have analyzed the binding residues in terms of “continuous stretch” in protein and RNA sequences and the results are presented in Figure 3a and b. The length of continuous binding residues is termed as a binding segment. We observed that the single residue segments are preferred uniformly by all the organisms followed by two-residue segments in proteins, which is consistent with our previous analysis on non-redundant set of protein-RNA complexes [4].

At the RNA level, most of the organisms prefer single nucleotide segments for binding with proteins. The preference of occurrence is approximately 30% in RNA whereas it is about 70% in proteins. The binding segments with more than two residues are observed in 70% of the binding sites in RNA. *E. coli* prefers to have binding segments with the length of 3, 4, 5 and more than 6 nucleotides whereas its preference is less for 2 and 6-residue segments. *H. sapiens* and *S. cerevisiae* have 20-25% of their binding sites in 2-residue segments and 10-15% have long stretch of binding sites with more than six nucleotides. Archaea has 25% of binding sites in 3-residue segments followed by 4 and 5-residue segments. These results reveal that the binding behavior of different organisms varies within the binding segments also for protein-RNA complexes and the observation was found to be statistically significant (p = 0.0347).

### Binding motifs in protein-RNA complexes from different organisms

The information obtained about the preference of binding site residues and nucleotides has been used to identify the potential motifs in protein and RNA for binding. We have computed the probability of all the possible tripeptides and trinucleotides that are involved in binding in different organisms. We noticed that some of the motifs are unique in the considered organisms as reported in the literature [42]. All the tripeptides NYV in *H. sapiens* and *S. cerevisiae* are involved in binding. In addition, tripeptide IQK has the probability of 100% and 80% for binding with RNA in *H. sapiens* and *S. cerevisiae*, respectively. In archaea, the tripeptides RRS and LKE have the probability of 100% and 75%, respectively in the binding sites. The total number of binding site residues in *E. coli* and *T. thermophilus* are less and hence are excluded in the analysis. At the RNA level ACA, GGU and UGU are preferred in *E. coli* whereas all the trinucleotides UUU in *H. sapiens* and *S. cerevisiae* are observed to be binding with proteins.

### Preference of dipeptides in the vicinity of binding sites

We have analyzed the preference of neighboring residues around the binding sites in protein-RNA complexes using the occurrence of dipeptides adjacent to the binding sites and their respective occurrences in the whole protein. The computations have been done using all possible 400 pairwise combinations of amino acid residues for the two categories, (i) *B (where '*'refers to any residue and B refers to the binding residue) and (ii) B*, and the preferred residue-pairs with the probability of more than 75% in any one of the organisms are presented in Tables 2 and 3. We noticed that few residue pairs (*B) are specific to a particular organism such as Cys-His in *H. sapiens*, Gly-Arg, Ser-Lys and Glu-Val in archaea (Table 2). Similar observation is also noticed in B* and specifically Val-Lys and His-Pro were observed in archaea (Table 3). This analysis reveals that the binding residue pairs are unique especially in archaea. On the other hand, several residue pairs are common for two to three organisms. For example, Ser-Asn has high preference in *E. coli*, *H. sapiens* and thermophiles, Asn-Tyr in *H. sapiens* and *S. cerevisiae* in *B. For B*, Tyr-Val is preferred in *E. coli*, *H. sapiens* and *S. cerevisiae*, His-Pro in *E. coli*, *H. sapiens* and archaea. These preferred residues pairs can be effectively used for identifying the binding sites in protein-RNA complexes. Further, we have examined the statistical significance of the data and the p-values of *B and B* are 3.6 × 10^−12^ and 1.2 × 10^−9^, respectively.

**Table 2:** Preferred residue pairs (*B) for binding with RNA

*B	**Probability (%)**				
	***E. coli***	***H. sapiens***	***S. cerevisiae***	**Thermophiles**	**Archaea**
Ala-Leu	75.0	0.0	0.0	20.0	0.0
Ser-Asn	100.0	100.0	37.5	75.0	0.0
Thr-Tyr	100.0	66.7	0.0	75.0	100.0
Cys-His	0.0	75.0	0.0	0.0	0.0
Gly-Thr	100.0	75.0	66.7	37.5	100.0
Asn-Tyr	0.0	100.0	100.0	0.0	0.0
Ile-Gln	0.0	100.0	72.7	0.0	0.0
Ser-Arg	0.0	40.0	100.0	100.0	57.1
Arg-Gly	0.0	50.0	0.0	75.0	50.0
Ala-His	0.0	0.0	0.0	80.0	75.0
Asn-Arg	50.0	0.0	0.0	100.0	100.0
Asn-Lys	0.0	25.0	25.0	100.0	75.0
Lys-Thr	50.0	0.0	0.0	100.0	100.0
Gly-Arg	30.0	50.0	0.0	38.5	71.4
Ser-Lys	50.0	36.4	0.0	0.0	75.0
Thr-Pro	0.0	100.0	0.0	50.0	75.0
Val-Lys	100.0	33.3	50.0	50.0	75.0
Glu-Val	0.0	0.0	0.0	16.7	80.0

**Table 3:** Preferred residue pairs (B*) for binding with RNA

B*	**Probability (%)**				
	***E. coli***	***H. sapiens***	***S. cerevisiae***	**Thermophiles**	**Archaea**
Arg-Gln	75.0	0.0	50.0	0.0	100.0
Gln-Lys	0.0	60.0	71.4	100.0	50.0
Arg-Ile	0.0	100.0	33.3	0.0	83.3
Arg-Val	100.0	100.0	42.9	41.7	0.0
Asn-Tyr	0.0	100.0	100.0	0.0	75.0
Tyr-Val	100.0	100.0	75.0	0.0	0.0
Ser-Asn	0.0	100.0	28.6	75.0	0.0
Asp-Arg	0.0	0.0	0.0	80.0	25.0
Gln-Ala	0.0	0.0	0.0	100.0	75.0
Ser-Arg	0.0	0.0	100.0	0.0	75.0
Val-Lys	0.0	0.0	0.0	50.0	75.0
Arg-Arg	28.6	0.0	0.0	25.0	100.0
His-Pro	75.0	100.0	0.0	50.0	100.0

### Preference of interacting amino acid-nucleotide pairs

We have analyzed the preference of interacting residues/nucleotides in proteins and RNA by calculating their pair preferences at the binding sites. The preferences of amino acid-nucleotide pairs have been converted into energy potentials to understand the preferred and avoided residue-nucleotide pairs for binding. The pairs, which have the values of less than −0.5 are considered as preferred and the ones with greater than 0.5 are treated as avoided. We noticed that the preferred and avoided amino acid residues are specific to interact with RNA and in different organisms (Table 4). The preferred residue-nucleotide pairs are Gly-C, Ala-C, Ser-C, Tyr-C, Asn-C and Leu-U in *E. coli*, Val-A, Cys-A, Trp-G and His-U in *H. sapiens*, Tyr-A, Gln-A and Met-G in *S. cerevisiae*, Val-C, Leu-C, Ile-C, Trp-C and Trp-U in thermophiles and Pro-C, Ile-U, Met-U, Ser-U, Cys-U and Glu-U in archaea. This analysis reveals that the preferred amino acids show inclination towards pairing with cytosine in *E. coli* and with adenine in *H. sapiens* and *S. cerevisiae*. Thermophiles and archaea show high preference to interact with cytosine and uracil, respectively. The potentials for all the possible 80 pairs are given in Additional file 1: Table S1 and the data are statistically significant (p = 0.0126). The potentials developed in this work will be useful for predicting the binding specificity of protein-RNA complexes belonging to different organisms.

**Table 4:** **Preferred and avoided amino acid-nucleotide pairs in different organisms**

**Organism**	**Prefered**	**Avoided**
*E. coli*	Gly-C, Ala-C, Ser-C, Tyr-C, Asn-C, Leu-C	Tyr-A, Phe-C, Met-C, Pro-U, Thr-U, Gln-U
*H. sapiens*	Val-A, Cys-A, Trp-G, His-U	Glu-A, Asn-G, Lys-G, Phe-C, Ser-c, Thr-C, Asp-C, Val-U, Leu-U, Ser-U
*S. cerevisiae*	Tyr-A, Gln-A, Met-G, Phe-C, Met-C, Thr-C, Phe-U	Leu-A, Gly-G, Arg-G
Thermophiles	Val-C, Leu-C, Ile-C, Trp-C, Trp-U	Ala-A, Val-A, Gln-A, Asp-G, Phe-C, Met-C, Ser-C, Ala-U, Phe-U, Ser-U, Asn-U, Asp-U
Archaea	Trp-A, Lys-A, Ala-G, Val-G, Ile-G, Pro-C, Ile-U, Met-U, Ser-U, Cys-U, Glu-U	Leu-A, Phe-A, Gln-G, Ala-C, Leu-C, Ile-C, Met-C, Thr-C, Tyr-C, Asn-C, Phe-U

### Case study

**Figure 4:**
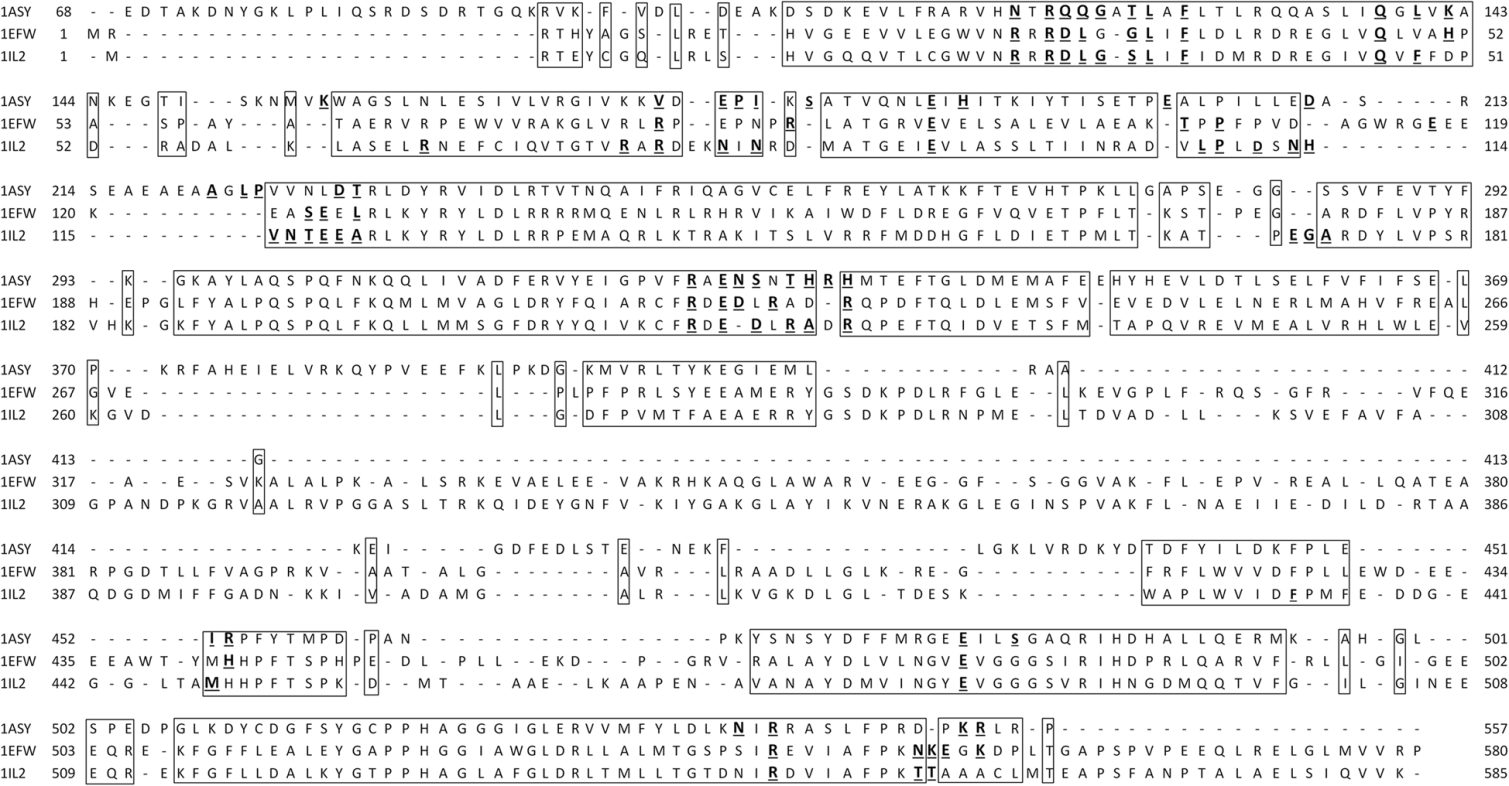
**Structure based sequence alignment of aspartyl tRNA complexes, 1ASY, 1EFW and 1IL2.** The structurally conserved regions are shown in boxes. The interacting residues are highlighted with bold letters.

We have extensively studied the variation of binding site residues in different organisms for each protein-RNA complex and the normalized binding propensities of 20 amino acid residues for a typical complex, AspRS-tRNA^Asp^ from *E. coli*, *T. thermophilus* and *S. cerevisiae* are shown in Table 5. We observed that the binding mode and binding site residues are distinct in these organisms. Phe prefers to be in the binding sites in *E. coli* whereas Gly is prefered in *T. thermophilus* and Pro, Met and Thr are prefered in *S. cerevisiae*. Although Asn, Glu and Arg show preference to be at the interface in all the organisms, the strength is different among them. The preference of Arg was higher in *E. coli* and *T. thermophilus* than Lys whereas an opposite trend was observed in *S. cerevisiae*. The structure based sequence alignment of AspRS from three different organisms is shown in Figure 4. We observed that the binding site residues, binding mode and binding segments are different among the three different organisms in the considered complex. The analysis of binding segments showed a similar trend at the protein level however the behavior is different in RNA among different organisms. Single nucleotide segments accommodated 67% of the binding sites in *T. thermophilus* whereas only 33% of the binding sites have single nucleotide segments in *E. coli*.

**Table 5:** **Propensity of amino acid residues in three different organisms of aspartyl tRNA synthetase**

**Amino acid**	***E. coli***	***T. thermophilus***	***S. cerevisiae***
Gly	0.65	1.12	0.39
Ala	0.75	0.00	0.35
Val	0.35	0.00	0.39
Leu	0.73	0.64	0.93
Ile	0.00	0.00	0.90
Pro	0.52	0.62	1.46
Phe	1.45	0.84	0.83
Trp	0.00	0.00	0.00
Met	0.67	0.00	1.17
Ser	0.70	0.00	1.40
Thr	1.32	1.58	1.40
Cys	0.00	0.00	0.00
Tyr	0.00	0.00	0.00
Gln	0.74	1.58	1.37
Asn	2.68	4.20	3.11
Asp	0.92	0.93	0.75
Glu	2.16	1.88	1.33
Lys	0.00	1.15	1.30
Arg	3.01	2.48	1.09
His	1.56	2.10	0.97

The mode of recognition for protein-RNA complexes belonging to different organisms has been further studied with a typical complex, AspRS-tRNA^Asp^ using molecular dynamics simulations as described in the Methods section. The overall binding free energy for AspRS-tRNA^Asp^ complexes from *E. coli*, *T. thermophilus* and *S. cerevisiae* are −212 ± 19.9 kcal/mol, −116.6 ± 14.3 kcal/mol and −190.9 ± 12.6 kcal/mol, respectively. The free energy is remarkably higher for *T. thermophilus* compared with its homologues indicating its low affinity for binding. This might be due to the fact that the thermophiles mainly account for their stability and are capable to sustain at high temperature. This has been confirmed with a large conformational change in the anti-codon loop of the complex from *E. coli*.

Further, T. thermophilius has half the number of binding sites compared with *S. cerevisiae* and *E. coli*, indicating its major role on stabilizing the complex. The energetic analysis shows that 17, 14 and 23 residues, respectively in *E. coli*, *T. thermophilus* and *S. cerevisiae*, potentially bind with RNA with a free energy of less than −3 kcal/mol. The hydrogen bond analysis shows the presence of 2069, 2131 and 1826 interactions in *E. coli*, *T. thermophilus* and *S. cerevisiae* respectively. Among them 114, 116 and 124 interactions are more stable with an occupancy of >80%. Specifically, 10 and 17 interactions strongly stabilize the AspRS-tRNA^Asp^ complexes of *E. coli* and *S. cerevisiae*, respectively while only 5 interactions were found at the interface in the case of *T. thermophilus*. It is due to the conformational fluctuation of the cognate tRNA, which leads to less number of hydrogen bonds in *T. thermophilus* than in other complexes. Conversely, the total number of interactions stabilizing the *T. thermophilus* RS (90) is higher than *E. coli* (71) and *S. cerevisiae* (64). We have also estimated the number of stabilizing residues in these three organisms using SRide server [43].We found that the *T. thermophilus* has the highest number of 51 stabilizing residues followed by *E. coli* (42) and *S. cerevisiae* (34).

In addition, Table 6 provides the positional relationship of binding site residues with high affinity and it reveals the difference in recognition mechanism in the three organisms. These high affinity binding residues span different RNA binding regions of AspRS such as anti-codon binding domain, hinge region, catalytic and insertion domains. The tRNA^Asp^ binding residues at anti-codon binding region are conserved among the three organisms and showed less variations. However, significant variation has been observed in the hinge and catalytic domains. Mechanism of recognition of tRNA by the RNA synthetase begins with binding of anti-codon bases with the hydrophobic residues at anti-codon binding domain of the protein. These variations in hinge and catalytic domains among different organisms dictate their unique mode of recognition of AspRS.

**Table 6:** **List of residues from different regions of AspRS strongly binding with tRNAAsp**

***E. coli***	***T. thermophilus***	***S. cerevisiae***	**Region**
*R26*	**R27**	*N117*	Anti-codon binding
**R28**	**R29**	**R119**	
**L30**	**L31**	**Q121**	
**L33**	**L34**	**L125**	
**G31**	-	-	
**F35**	**F36**	**F127**	
**N84**	**N82**	**I179**	
**S32**	*G33*	*T124*	
*D86*	**R84**	**K180**	
**E93**	**E91**	**E188**	
*Q46*	**Q47**	*Q138*	
-	-	L223	
**R64**	**R64**	*N161*	
*R78*	**R78**	*V175*	
-	-	**P224**	
**V107**	-	**V226**	
**T117**	-	**N227**	Hinge region
-	-	**L228**	
-	**R115**	-	
*A120*	*L126*	**T230**	
**R217**	-	**R325**	Catalytic domain
**R222**	*A229*	**T331**	
*D224*	-	**R333**	
R225	*R231*	**H334**	
*F229*	*F235*	**F338**	
*I343*	**R343**	-	Insertion domain
-	-	**T424**	Catalytic domain
-	-	**K428**	
**T558**	**K552**	-	
*R537*	*R531*	**R531**	
**R549**	*R543*	*R544*	
*A560*	*G554*	K553	
**T557**	*N551*	*D551*	
*A561*	*K555*	**R554**	

Bold face indicates the set of residues strongly interacting with tRNA^Asp^. The equivalent residues from other sources, which are not interacting with tRNA^Asp^ are italics"-" indicates gap or no equivalent residues.

The organism specific recognition of protein-RNA complexes may be attributed with the following perspectives: (i) every stage of RNA metabolism is driven by binding of RNA binding proteins (RBPs) through RNA binding domains. In general, RBPs are structurally diverse as the complexity of the genome is increased during evolution and they are recruited at different stages during transcription and translation processes [44,45], (ii) horizontal gene transfer [46] and (iii) RBPs acquire evolutionarily conserved structures and they show difference at sequence level in each subfamily. As discussed in the case study, these differences influence the mode of binding with its tRNA substrate. This may be further examined with detailed analysis on various pairs of protein-RNA complexes.

## Conclusions

We have investigated the organism specific recognition of protein-RNA complexes based on various sequence and structure based features such as binding propensity, preference of residues at conserved positions, binding segments, binding motifs, neighboring residues and interacting amino acid-nucleotide pairs. The results showed that the residue and nucleotide preferences are distinct in different organisms. The preference of amino acid residue pairs obtained in the present work will be useful for predicting the binding sites of RNA binding proteins. We have developed amino acid-nucleotide pair potentials for different organisms, which can be used for predicting the binding specificity of protein-RNA complexes. The molecular dynamics simulations studies on a typical complex, AspRS-tRNA^Asp^ showed the specific mode of recognition as well as preferred binding sites in different organisms. These results provide deep insights to understand the recognition of protein-RNA complexes belonging to different organisms.

## Reviewers’ comments and response

### Reviewer #1: Professor Sandor Pongor

In this work, the authors have analyzed the binding specificity of 18 sets of homologous protein-RNA complexes belonging to different organisms. This is a different approach from the traditional analysis with non-redundant datasets. The investigations have been carried out on various sequence and structure based features as well as molecular dynamics simulations. The results showed the similarities and differences between different organisms in the same complex. Further, distinct modes of recognition have been revealed with a typical example using MD simulations and energy calculations. The work would have further implications on understanding the recognition mechanism of protein-RNA complexes from different organisms.

1. It has been mentioned that the potentials for amino acid-nucleotide pairs derived for different sets of organisms would be helpful for predicting the binding specificity. However, the data are not shown. The potentials should be given in supplementary information.

Authors’ response: *Amino acid-nucleotide pair potentials are given in supplementary Table S1.*

2. The stability of aspartyl tRNA synthetase from E. coli, T. thermophiles and S.cerevisiae could be discussed with stabilizing residues in these complexes.

Authors’ response: *The stability has been discussed with the number of stabilizing residues.*

3. The cutoff used to select the preferred and avoided residues in Table 3 may be given.

Authors’ response: *Values less than -0.5 are considered as preferred and greater than 0.5 as avoided amino acid-nucleotide pair preference.*

### Reviewer #2: Professor Narayanaswamy Srinivasan

Gromiha et al have performed comparative analysis of 3-D structures of homologous proteins bound to RNA. They have analysed number of RNA binding sites, amino acid residues which are involved in RNA recognition, segments in proteins and RNAs involved in recognition of each other etc. The most important new feature of this analysis is to view these structural attributes in terms of organisms. This led to recognition of organism-dependent features in protein-RNA complexes. This is a new and important finding. Though physicochemical nature of the binding sites determine the specificity and stability of the complexes, learning from this manuscript provides a new dimension to protein-RNA recognition based on the type of the organism. I think a round of revision is needed before this work may be published.

1. The most important outcome of this work is the "organism-dependent" features of protein-RNA complexes. This must be ensured by statistical significance tests. I hope the observed frequencies of various features, such as amino acids involved in RNA binding, and the size of the dataset will permit authors to perform meaningful statistical significance tests, Data presented in most of the Tables and Figures must be subjected to statistical significance tests. In my view this is a crucial addition to be made in the revised version.

Authors’ response: *We have performed statistical significance tests for the results presented in Tables and Figures using ANOVA, wherever possible. The p-values are less than 0.05 for most of the data, which validates the results.*

2. I understood that dataset formation involved groups of protein-RNA complex structures with proteins being homologous. What about RNA sequences in each group? Can the observed differences in preferred amino acids which recognize RNA be explained in terms of base sequence differences in bound RNA?

Authors’ response: *We have evaluated the influence of RNA base sequence on binding propensity of amino acid residues in nucleotides. The lengths of RNA sequences are almost similar in all the complexes and the sequence identity varies in the range of 40-100% in most of the considered complexes. We have analyzed the nucleotide sequences at the binding sites in different pairs of protein-RNA complexes and observed that the binding preference is similar in all the nucleotides. Further, the change in propensities of amino acid residues is not uniform with similar change in nucleotides. These analyses reveal that the influence of base sequence is not appreciable compared with amino acid sequences of protein-RNA complexes from different organisms. However, this effect can be extensively studied using systematic analysis on mutations and molecular dynamics simulations for deriving any conclusions.*

3. While the manuscript is well organized, it requires sorting out typos and refinement throughout the manuscript. For example, in the Abstract authors mention "We have found that the mesophilic organisms have more number of binding sites than thermophiles and....". I am sure authors mean proteins of mesophilic and thermophilic organisms not organisms themselves. In another place in the Abstract authors mention "Proteins prefer to bind with RNA using a single residue in.....". It is not clear if authors mean segments with a single residue or single segment.

Authors’ response: *The language corrections have been carried out.*

### Reviewer #3: Dr Gajendra Raghava

In this manuscript authors analyzed Protein-RNA complexes to understand RNA binding in different organism. They obtained Protein-RNA complexes from different organisms and compute binding preference of residues in protein and nucleotides in RNA. Their observation is interesting that different residue are preferred in different organism, similarly nucleotide preference is also different in different organism. This reviewer have following point for authors.

1. What is impact of crystallization conditions particularly temperature on RNA binding, authors should examine this issue. Authors should also examine whether Protein-RNA complexes were expressed in their host or not.

Authors’ response: *We have checked the crystallization conditions, and found that more than 90% of structures in the dataset have the same temperature (100 K). In all the cases, the expression organism is E. coli*.

2. Deviation in preference of residues among proteins belongs to same organism, similarly variation in nucleotide preferences among RNAs belongs to same organism should be examined. Standard deviation in residue/nucleotide plot may provide this information.

Authors’ response: *Deviations are included in all the figures.*

3. Significance should be calculated to understand whether preference is really different.

Authors’ response: *We have performed statistical significance tests for the results presented in Tables and Figures using ANOVA, wherever possible. The p-values are less than 0.05 for most of the data.*

4. If possible, authors should provide reasons why binding is different in Protein-RNA complexes belongs to different organisms.

Authors’ response: *(i) Every stage of RNA metabolism is driven by binding of RNA binding proteins (RBPs) through RNA binding domains. In general, RBPs are structurally diverse as the complexity of the genome is increased during evolution and they are recruited at different stages during transcription and translation processes* [44,45]*, (ii) horizontal gene transfer* [46] *and (iii) in each subfamily, RBPs acquire evolutionarily conserved structures and they show difference at sequence level. As discussed in the case study these differences influence the mode of binding with its tRNA substrate. This may be further examined with detailed analysis on various pairs of protein-RNA complexes.*

## Additional file

Additional file 1: Table S1. Amino acid-nucleotide pair potentials in different organisms.

## Abbreviations

RNA: Ribonucleic acid
PDB: Protein data bank
FRET: Fluorescence resonance energy transfer
PSSM: Position specific scoring matrices
tRNA: Transfer RNA
AspRS: Aspartyl tRNA synthetase
tRNAAsp: Aspartyl tRNA
RMSD: Root mean square deviation
AMBER: Assisted Model Building with Energy Refinement
PME: Particle Mesh Ewald
MM-GB/SA: Molecular Mechanics-Generalied Born/Surface Area
